# Material Properties and Cell Compatibility of Photo-Crosslinked Sericin Urethane Methacryloyl Hydrogel

**DOI:** 10.3390/gels8090543

**Published:** 2022-08-29

**Authors:** Safaa Kader, Esmaiel Jabbari

**Affiliations:** 1 Biomaterials and Tissue Engineering Laboratory, Department of Chemical Engineering, Swearingen Engineering Center, Rm 2C11, University of South Carolina, Columbia, SC 29208, USA; 2 Department of Pathology and Forensic Medicine, College of Medicine, Al-Nahrain University, Baghdad 10006, Iraq

**Keywords:** natural hydrogel, silk sericin, sericin urethane methacryloyl, cell encapsulation, mesenchymal stem cells

## Abstract

There is a need to develop novel cytocompatible hydrogels for cell encapsulation and delivery in regenerative medicine. The objective of this work was to synthesize isocyanato ethyl methacryloyl-functionalized sericin and determine its material properties as a natural hydrogel for the encapsulation and delivery of human mesenchymal stem cells (MSCs) in regenerative medicine. Sericin extracted from silk cocoons was reacted with 2-isocyanatoethyl methacrylate (IEM) or methacrylic anhydride (MA) to produce sericin urethane methacryloyl (SerAte-UM) or sericin methacryloyl (SerAte-M, control) biopolymers, respectively. The hydrogels produced by photo-crosslinking of the biopolymers in an aqueous solution were characterized with respect to gelation kinetics, microstructure, compressive modulus, water content, degradation, permeability, and viability of encapsulated cells. The secondary structure of citric acid-extracted sericin was not affected by functionalization with IEM or MA. SerAte-UM hydrogel was slightly more hydrophilic than SerAte-M. The gelation time of SerAte-UM hydrogel decreased with an increasing degree of modification. The photo-polymerized SerAte-UM hydrogel had a highly porous, fibrous, honeycomb microstructure with an average pore size in the 40–50 µm range. The compressive modulus, swelling ratio, and permeability of SerAte-UM hydrogel depended on the degree of modification of sericin, and the mass loss after 21 days of incubation in aqueous solution was <25%. Both SerAte-UM and SerAte-M hydrogels supported viability and growth in encapsulated MSCs. The SerAte-UM hydrogel, with its higher hydrophilicity compared to SerAte-M, is promising as a matrix for encapsulation and delivery of stem cells in tissue engineering.

## 1. Introduction

Hydrogels, due to their high water content and diffusivity of peptides and proteins, are attractive as a matrix for encapsulation and delivery of cells for the regeneration of functional tissues [1,2,3,4,5]. Natural hydrogels mimic the biological extracellular matrix (ECM) due to their inherent biocompatibility, biodegradability, and cell-binding capability [6,7,8,9]. As natural hydrogels degrade enzymatically in response to cell secretions, they are effective as injectable carriers for the delivery of growth factors in regenerative medicine [10,11]. Furthermore, due to their hierarchical structure, natural hydrogels enable spatial patterning of the seeded cells to regenerate the desired functional tissue [12,13]. Scaffolds based on natural hydrogels such as collagen, gelatin, alginate, keratin, and chitosan have demonstrated excellent cytocompatibility and spatially-organized cell–matrix interactions to regenerate functional tissues [9,14,15,16,17]. There is a need to develop novel natural hydrogels with improved and tunable mechanical properties for the regeneration of load-bearing soft tissues. Sericin, the most abundant protein in silk, has received considerable attention as a scaffold for tissue regeneration due to its non-mutagenicity, non-toxicity, and cytocompatibility [18,19,20]. Notably, sericin is rich in hydroxyl, amino, and carboxyl groups for functionalization to tune its chemical and mechanical properties for regeneration of the desired soft tissue [21]. Sericin has been functionalized with methacryloyl groups and crosslinked by photopolymerization to produce hydrogel scaffolds for regeneration of cartilage [22], cardiac [23], and sciatic nerve [24] tissues, as well as wound healing [25]. It has been shown that the physiochemical and mechanical properties of hydrogel networks are significantly affected by slight changes in the chemistry of the functional groups used for crosslinking [26]. In this respect, the functionalization of polyethylene glycol-based macromers with urethane methacryloyl groups, as opposed to methacryloyl, improved the physiochemical and mechanical properties of the resulting hydrogel, which was attributed to increased secondary interaction between the biopolymer chains due to hydrogen bonding [26]. There is a need to improve the properties of sericin-based hydrogel scaffolds using novel functionalization chemistries.

**Figure 1:**
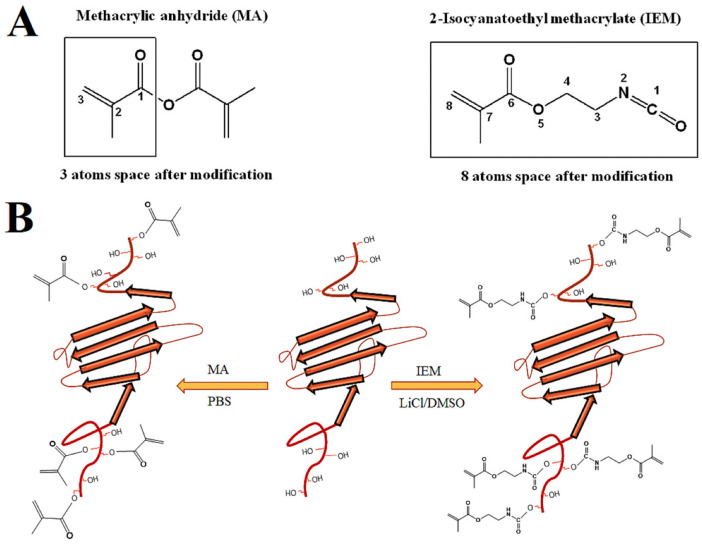
(**A**) Chemical structure of methacrylic anhydride (MA) and isocyanatoethyl methacrylate (IEM) showing the three- and eight-atom linkers after modification, respectively. (**B**) Modification of sericin through its hydroxyl groups with MA in PBS and with IEM in DMSO with LiCl as the catalyst to form hydrogels through photo-polymerization.

The objective of this work was to functionalize sericin with urethane methacryloyl groups to produce a sericin urethane methacryloyl (SerAte-UM) hydrogel and compare its properties with that of sericin methacryloyl (SerAte-M) hydrogel. Sericin was extracted from silk cocoons using the acid degumming method [27]. Next, the extracted sericin was modified with 2-isocyanatoethyl methacrylate (IEM) or methacrylic anhydride (MA) and the functionalized macromers were crosslinked by photo-polymerization, as shown in Figure 1. The functionalized sericins were characterized using nuclear magnetic spectroscopy (NMR), infrared (IR) spectroscopy, circular dichroism (CD), and gel electrophoresis. Rheometry was used to characterize the gelation kinetics of the hydrogel precursor solutions. The SerAte hydrogels were characterized via water content, dye permeability, hydrolytic degradation, microstructure, adhesion and proliferation of human mesenchymal stem cells (hMSCs).

## 2. Materials and Methods

### 2.1. Materials

Bombyx Mori silk cocoons (A Quality) were obtained from Yarn Tree (Greenville, SC, USA). Diethyl ether, citric acid, lithium chloride (LiCl), dimethyl sulfoxide (DMSO), MA, and IEM were purchased from VWR (Bristol, CT, USA). Streptomycin (STM), penicillin (PEN), and glutamine (GLU) were purchased from MilliporeSigma (Burlington, MA, USA). Phosphate-buffer saline (PBS) and Alpha modified Eagle’s minimum essential medium (α-MEM) were purchased from GIBCO BRL (Grand Island, NY, USA). Trypsin and fetal bovine serum (FBS) were received from Invitrogen (Carlsbad, CA, USA) and Atlas Biologicals (Fort Collins, CO, USA), respectively. The stains 3,4,7,8-Tetramethyl-1,10-phenanthroline (TRITC)-conjugated phalloidin and 4,6-diamidino-2-phenylindole (DAPI) were purchased from EMD Millipore (Billerica, MA, USA). The Calcein-acetoxymethyl (Calcein-AM) and ethidium homodimer-1 (EthD) cell viability/cytotoxicity assays for imaging live and dead cells, respectively, were purchased from Molecular Probes (Life Technologies, Grand Island, NY). Dialysis tubes with molecular weight cut-off (MWCO) of 3.5 kDa were purchased from Spectrum Laboratories (Rancho Dominquez, CA, USA). Centrifugal filter tubes (Amicon Ultra-15) with MWCO of 30 kDa were purchased from MilliporeSigma. Bovine serum albumin (BSA) was received from Jackson ImmunoResearch (West Grove, PA, USA). PicoGreen assay for quantification of dsDNA content of cells was purchased from Molecular Probes (ThermoFisher Scientific, Waltham, MA, USA).

### 2.2. Extraction of Sericin from Cocoons

Sericin was extracted from silk cocoons using the previously reported acid extraction method with modifications [28]. Briefly, silk cocoons were cleaned by soaking in ether, washed in soapy water, and rinsed in deionized (DI) water. The cleaned cocoons were dried and chopped into small pieces approximately 1 cm^2^ in size. Next, the chopped cocoons (9.0 g) were boiled in 160 mL of 1.25 *w*/*v* citric acid aqueous solution for 30 min for sericin extraction. After boiling, the insoluble fraction was removed by centrifugation at 4400 rpm, and the filtrate was transferred to a dialysis tube (3.5 kDa MWCO) and dialyzed against DI water at neutral pH for three days at ambient conditions with a change of DI water every 6 h. The dialyzed solution was lyophilized to dryness and stored at −20 °C. The extracted sericin was dried in vacuum at 50 °C for 24 h to remove any residual water in the protein that could interfere with the reaction of protein with IEM in anhydrous DMSO.

### 2.3. Synthesis of Sericin Urethane Methacryloyl (SerAte-UM)

SerAte-UM was synthesized using the following approach, as described previously [29]. Briefly, 1.0 g of extracted sericin (2 wt%) was dissolved in a solution of anhydrous LiCl/DMSO at 60 °C for 45 min under a nitrogen atmosphere. Next, IEM was added to the solution dropwise at different fold excess molar ratios relative to the serine in sericin, ranging from 7:1 to 20:1 IEM:serine as shown in Table 1, and the reaction was allowed to continue for 24 h. After reaction completion, the product was collected and dialyzed against DI water for three days to remove the unreacted IEM, followed by centrifugation to precipitate the product. The precipitate was flash-frozen in liquid nitrogen and lyophilized, and the resulting powder was stored at −20 °C. SerAte-M, as the control sample, was synthesized using the following approach described previously [25]. Briefly, 1.0 g of extracted sericin was dissolved in 7.0 mL PBS (pH 8.5) at 35 °C for 2 h. Next, MA in PBS (10 mL) was added to the solution dropwise at the same fold excess molar ratios as for IEM (see Table 1) and the reaction was allowed to continue overnight under stirring at ambient conditions. After the reaction, the product was dialyzed against DI water for three days, lyophilized, and stored at −20 °C.

### 2.4. Characterization of SerAte-UM and SerAte-M

The chemical structures of SerAte-UM and SerAte-M were characterized via nuclear magnetic resonance spectroscopy (^1^H-NMR), attenuated total reflection (ATR) infrared spectroscopy, and circular dichroism (CD). The ^1^H-NMR experiments were carried out using a Bruker DMX-3000 spectrometer (Billerica, MA, USA). The sericin samples were dissolved in deuterium oxide (D_2_O) at a concentration of 5 mg/mL. The ATR-IR spectra of the samples were recorded in powder form using a PerkinElmer 100 spectrometer with a diamond ATR cell attachment in the spatial frequency range of 4000–600 cm^−1^. The CD spectra of the samples were recorded in PBS at a concentration of 0.1 mg/mL using a JASCO J815 spectropolarimeter (Essex, UK) at ambient conditions. The molecular weight of sericin before and after modification was determined via sodium dodecyl sulfate polyacrylamide gel electrophoresis (SDS-PAGE). The extracted and modified sericin samples were dissolved in 2× electrophoresis buffer (BioRad, Hercules, CA, USA) containing 5 wt% 2-mercaptoethanol, and the proteins were separated using a vertical slab gel electrophoretic system with a 4–20% stacking gel. The electrophoresis experiments were performed at a voltage of 100 V and a current flow of 15 mA for 80 min. The proteins were stained with Coomassie brilliant blue R-250 (BioRad) for 1 h and de-stained with DI water according to the supplier’s instructions. The microstructure of the SerAte hydrogels was imaged using a VEGA3 SBU variable pressure scanning electron microscope (SEM; Tescan, Kohoutovice, Czech Republic) at an accelerating voltage of 8 keV. After immersion in liquid nitrogen, the freeze-dried samples were cut with a surgical blade to expose a freshly cut surface. The cut surface was coated with gold using a Denton Desk II sputter coater (Moorestown, NJ, USA) at a current of 20 mA for 75 s and imaged with the SEM.

### 2.5. Gelation and Rheological Measurements

The SerAte hydrogel precursor solutions were crosslinked by radical polymerization initiated by Irgacure 2959 (CIBA, Tarrytown, NY, USA) photoinitiator, as described previously [9]. Based on our previous results, the initiator concentration of 0.75 wt%, based on the macromer weight, was used in the hydrogel precursor solution to minimize cell toxicity and optimize the extent of gelation [30]. Briefly, the initiator solution (0.75 wt%) was added to an aqueous solution of the SerAte sample (10 mg/mL) with vortexing and the resulting hydrogel precursor solution was loaded onto the Peltier plate of an AR-2000 (TA Instruments, New Castle, DE, USA) rheometer. A 20 mm clear acrylic geometry at a gap distance of 500 µm was used to initiate polymerization of the gel precursor solution through UV irradiation, as we previously described [9]. The assembly was wrapped in aluminum foil to maximize UV exposure to the sample for crosslinking. A sinusoidal shear strain with 1 Hz frequency and 1% strain was applied at ambient conditions while the sample was irradiated with a BLAK-RAY 100-W mercury long-wavelength (365 nm) UV lamp (Model B100-AP; UVP, Upland, CA, USA). The storage (G′) and loss (G″) moduli of the sample were recorded with irradiation time. The time at which the curve for G′ crossed the curve for G″ was defined as the gelation time. For measurement of compressive modulus, the SerAte hydrogel precursor solution was transferred to a Teflon mold (5 cm × 3 cm × 750 nm), the mold was covered with a transparent glass cover, and the assembly was irradiated with UV for 2–3 min. Disk-shaped samples cut from the crosslinked hydrogels were loaded onto the Peltier plate and subjected to a uniaxial compressive force at a displacement rate of 7.5 µm/s. The slope of the linear fit to the stress–strain curve for a 10% strain was taken as the compressive modulus, as we previously described [9].

### 2.6. Swelling and Permeability Measurements

For measurement of swelling, each hydrogel sample was dried as described previously [9] and the dry weight (w_d_) was recorded. Next, the sample was immersed in 2 mL of PBS at ambient conditions, and the sample weight (w_s_) was recorded with incubation time. At each time point, the sample was removed, the surface water was wiped off with a Whatman filter paper, and the sample weight was recorded. Finally, the equilibrium sample weight (w_es_) was recorded when there was negligible change in sample weight after incubation. The swelling ratio of the hydrogel sample was calculated as the difference between the swollen and dry weights divided by the dry weight (w_s_–w_d_/w_d_) and the equilibrium swelling ratio was calculated as the difference between the equilibrium swollen and dry weights divided by the dry weight (w_es_–w_d_/w_d_). For measurement of permeability, each hydrogel sample was immersed in 2 mL of PBS containing 5 μg/mL fluoresceinamine isomer I dye (MilliporeSigma) and the amount of fluorescent dye permeated through the sample was measured with incubation time at ambient conditions. At each time point, the fluorescent intensity of the incubation medium was measured with a Spectramax (Molecular Devices, San Jose, CA, USA) microplate reader at excitation/emission wavelength of 490/520 nm. At each time point, the amount of permeated dye was determined by subtracting the amount remaining in the incubation medium at time *t* from the initial amount. The dye concentration in the hydrogel was calculated by dividing the amount of permeated dye by the volume of the hydrogel sample.

### 2.7. Hydrogel Degradation

For degradation measurements, disk-shaped hydrogel samples were dried, and their dry weights (w_d,0_) were recorded as described above. Next, each of the dried samples was incubated in 2 mL of PBS at ambient conditions. At each time point, a sample was removed from the incubation medium, washed with DI water, and dried in vacuum for 2 h at 40 °C, and the dry weight was measured (w_d_). This process was repeated for all time points. The measured dry weight at each time point was subtracted from the initial dry sample weight and normalized by dividing by the initial dry weight to find the fractional mass loss.

### 2.8. Cell Adhesion and Viability

Human MSCs (Lonza) were cultured in a basal medium composed of α-MEM medium supplemented with 16% FBS, 3 mM GLU, 100 units/mL PEN, and 100 µg/mL STM. After reaching 70% confluency, the MSCs were detached from the culture plate with 0.1% trypsin- 0.03% EDTA and sub-cultured at a ratio of 1:3 for <5 passages, according to the supplier’s instructions. Cell culture experiments were performed for up to three days to assess cell adhesion, viability, and proliferation. For cell adhesion experiments, 24-well culture plates were coated with a thin layer of SerAte precursor solution and the plates were irradiated with UV to form SerAte hydrogels. After washing the hydrogels with DI water followed by PBS, hMSCs were seeded on the surface of SerAte gel-coated well plates at a density of 5 × 10^3^ cells/cm^2^ and cultured in the basal medium for cell attachment. For immunofluorescent staining of adhered cells, well plates were washed with PBS and fixed with 4% paraformaldehyde (MilliporeSigma) at 4 °C for 30 min, permeabilized with 0.1% Triton X-100 and 100 mM glycine in PBS for 15 min, and blocked with 4% BSA and 0.5 mM glycine in PBS for 30 min. Next, the blocked samples were incubated with TRITC-conjugated phalloidin in a blocking buffer for 5 min at ambient conditions. After washing with PBS, the samples were counterstained with DAPI to image the cell nuclei. The stained samples were imaged with a Nikon Eclipse Ti-E inverted fluorescent microscope (Nikon, Melville, NY, USA) as previously described [9].

For cell proliferation experiments, hMSCs were added to the SerAte precursor solution at a density of 1 × 10^6^ cells/mL; the cell suspension was transferred into a mold and covered with a transparent glass plate, and the assembly was irradiated with UV to encapsulate the hMSCs in the hydrogel. After crosslinking, the cell-laden SerAte hydrogels were incubated in the basal medium for up to three days. At each of two time points (days 1 and 3), the hydrogel samples were washed with serum-free α-MEM followed by washing with PBS, and the encapsulated cells were lysed with 10 mM Tris supplemented with 0.2% Triton in PBS. The DNA content of the lysed samples was measured using the Quant-it PicoGreen assay as previously described [9]. The SerAte precursor solutions crosslinked without hMSCs were used as the negative control. For cell viability measurements, at each time point, the hydrogel samples were washed with PBS and incubated in serum-free medium containing 2 µM Calcein-AM and 4 µM EthD as live and dead cell stains, respectively, for 15 min. Next, the stained encapsulated cells were imaged with the inverted fluorescent microscope as previously described [9].

### 2.9. Statistical Analysis

Data are expressed as means ± standard deviation. All experiments were performed in triplicate. Significant differences between experimental groups were evaluated using a two-way ANOVA with replication test, followed by a two-tailed Student’s *t*-test. A value of *p* < 0.05 was considered statistically significant.

## 3. Results and Discussion

### 3.1. Characterization of SerAte-M and SerAte-UM

**Figure 2:**
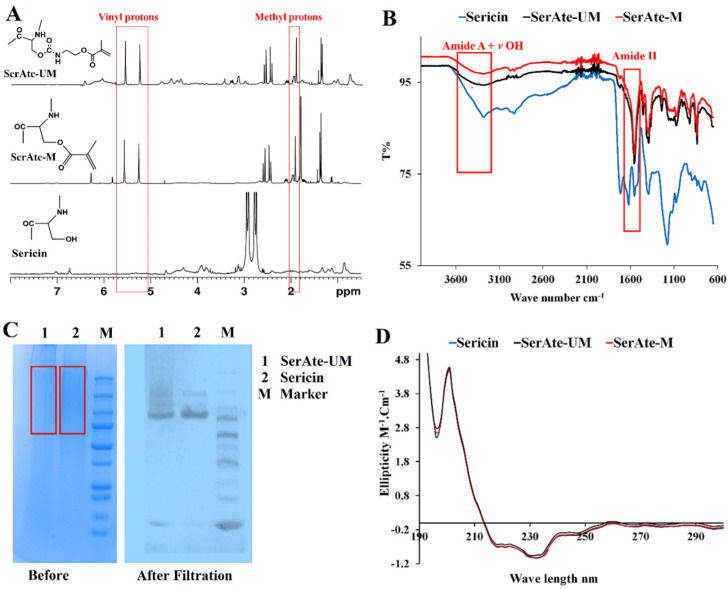
^1^H-NMR (**A**), IR (**B**), and CD (**D**) spectra of sericin (blue), SerAte-M (red), and SerAte-UM (black) in the wavenumber range of 4000–600 cm^−1^; SDS-PAGE (**C**) of SerAte-UM (1), sericin (2), and the standard protein MW markers (M) before and after filtration. The value of each standard MW for protein markers is provided in (**C**).

Sericin was extracted from silk cocoons by hydrolytic degumming using the citric acid method to maintain sericin’s natural properties, as previously described [21]. The treatment step with urea was not included in the citric acid method, as urea significantly affects the natural conformation of sericin. The ^1^H-NMR spectra of sericin, SerAte-M, and SerAte-UM are compared in Figure 2A. In the previous work by Lim et al. [31], sericin was extracted from silk cocoons using an alkali method, which is less soluble in aqueous solution compared with the sericin we extracted with citric acid. Due to the hygroscopic nature of our extracted sericin, a strong water peak was observed at 4.79 ppm, which had to be knocked down to see the entire sericin NMR spectrum. The NMR spectra of the samples were run with solvent pre-saturation, in which a long low-power pulse was applied to the solvent (water) resonance before the pulse sequence to knock down the solvent peak [32]. The solvent pre-saturation produced an out-of-phase residual peak in the NMR spectra of sericin, SerAte-M, and SerAte-UM, as shown in Figure 2A. The two peaks centered between 5.5–6.5 ppm in the NMR spectra of SerAte-M and SerAte-UM were attributed to the vinyl protons of methacrylate. The chemical shift centered around 3 ppm in the spectrum of sericin consisted of two resonances coupled to each other with a coupling constant of 16 Hz. This coupling constant is consistent with both hydrogens being on the same carbon atom. The presence of chemical shifts in the SerAte-M and SerAte-UM spectra centered at 1.9 ppm and 5.5–6.5 ppm corresponding to methyl and vinyl protons, respectively, confirmed the modification of sericin with methacryloyl and urethane methacryloyl groups. The degree of modification was calculated from the NMR spectra by comparing the area under the vinyl proton peaks of methacryloyl in SerAte-M or SerAte-UM to the area under the ethyl proton peaks associated with the serine and glycine residues (3.6–4.0 ppm) in sericin, as previously described [31]. The degree of modification for MA/IEM:sericin ratios of 7:1, 12:1, and 20:1 was 24 ± 2%, 31 ± 5%, and 40 ± 3%, respectively (Table 1).

The IR spectra of sericin, SerAte-M, and SerAte-UM are compared in Figure 2B. The absorption bands centered at 3270 cm^−1^, attributed to N–H bond symmetric stretching (amide A), was reduced after sericin modification. Similarly, the absorption band centered at 3400 cm^−1^, due to the O–H bond in sericin and water, was reduced after sericin modification [33]. The IR spectra of proteins show a strong signal in the 1700–1600 cm^−1^ region attributed to amide I vibration. However, there are several drawbacks to using this absorption band to study the secondary structure of proteins [32]. First, the OH vibrations of residual water molecules in the protein sample interfere with the amide I vibration, which needs to be subtracted. Second, the overlapping of absorption bands complicates band assignment to specific secondary structures. For example, the band in the 1650–1655 cm^−1^ could be assigned to either a random coil or an α-helical structure [32]. Conversely, the amide III bands in the 1220–1320 cm^−1^ region do not have the above complications, as interference with OH vibrations of water molecules and overlapping of bands arising from different secondary structures are less pronounced compared to the 1700–1600 cm^−1^ region [32]. Therefore, we compared the 1650–1700 cm^−1^ region of the spectra of sericin, SerAte-M, and SerAte-UM samples. The absorption bands centered at 1650 cm^−1^ (amide I, C=O stretching), 1530 cm^−1^ (amide II, N–H bending and C–N stretching), 1400 cm^−1^ (amide III, C–N stretching), and 1070 cm^−1^ (amide III, C–O stretching) decreased in the spectra of SerAte-M and SerAte-UM. The absorption bands for the acrylate group centered at 980 cm^−1^ (C=C–H), 3030 cm^−1^ (CH_2_=CH–CO), 1710 cm^−1^ and 1650 cm^−1^ (C=O), 1680 cm^−1^ (C–O), and 1620 cm^−1^ (C=C) increased in the spectra of SerAte-M and SerAte-UM. These changes in absorption bands indicated successful modification of sericin with methacryloyl or urethane methacryloyl groups.

The SDS-PAGE gel images of the extracted sericin and SerAte-UM are compared with the standard MW protein markers in Figure 2C (before filtration). The stained images showed a diffuse pattern of molecular weights in the range of 75–250 kDa for sericin and SerAte-UM, which implied some degradation of sericin during the extraction process. Next, the samples were filtered using the following procedure to obtain sericin and SerAte-UM samples with narrow molecular weights. Briefly, 12 mL of the sample was added to a centrifugal filter tube with MWCO of 30 kDa, spun twice at a maximum rotation speed of 4500× *g* for approximately 20 min to prevent protein aggregation, and the concentrated protein was recovered with a pipette. The SDS-PAGE gel images of the filtered samples are shown in Figure 2C. After filtration, two distinct bands at approximately 85 kDa and 150 kDa were observed in the SDS-PAGE gel image of sericin, which was consistent with the previously reported bands at approximately 60 kDa, 100 kDa, and 130 kDa [34]. In contrast, the filtered SerAte-UM sample showed a diffuse band in the molecular weight range of 85 kDa to 250 kDa, which was attributed to multiple modifications of sericin by IEM [35]. For crosslinking, the fractions with molecular weights > 250 kDa were discarded due to incomplete solubility in the aqueous solution.

The CD spectra of sericin, SerAte-M, and SerAte-UM in PBS at ambient conditions are shown in Figure 2D. The CD spectra of the samples displayed two shallow troughs centered at 217 nm and 230 nm and a peak centered at 197 nm, consistent with the native sericin CD spectrum [36]. The CD spectra indicated that the extracted sericin had a coil conformation containing a small amount of β-sheet secondary structure. The presence of this β-sheet structure in sericin was attributed to its high serine content, as previously reported [36]. There was no significant change in the CD spectrum of sericin after modification, implying that the secondary structure of sericin was not affected by modification with MA or IEM.

### 3.2. Kinetics of Gelation of SerAte-UM

**Figure 3:**
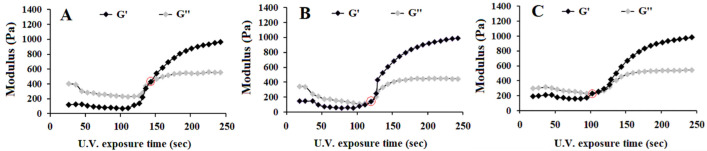
The effect of UV exposure time on the storage (G′) and loss (G″) moduli of 15 wt% SerAte-UM precursor solution with degree of modification of 24% (**A**), 31% (**B**), and 40% (**C**). The red circles show the gelation time at which G′ = G″.

The effect of UV irradiation time on the storage (G′) and loss (G″) moduli of SerAte-UM precursor solutions with 24%, 31%, and 40% degrees of modification are shown in Figure 3A–C, respectively. The UV exposure time at which the increasing G′ curve intersected the decreasing G″ curve was defined as the gelation time, as described previously [37]. The gelation time of the precursor solution decreased from 140 ± 15 s to 120 ± 10 s and 100 ± 10 s as the degree of modification increased from 24% to 31% and 40%, respectively. This decrease in gelation time was attributed to the higher density of reactive methacrylate groups in sericin-UM, which increased the rate of crosslinking between the sericin chains [37]. For cell encapsulation, the gelation time should be sufficiently short to reduce cell exposure to the photo-initiator and unreacted methacrylate groups in the reaction mixture [38]. It was reported that prolonged exposure of bone cells to functional methacrylate groups caused aseptic inflammation and tissue destruction in an ovine model of screw augmentation [39]. Furthermore, a high degree of modification of serine residues in sericin and over-crosslinking negatively affected cell spreading and attachment to the hydrogel matrix [40,41]. Therefore, based on the gelation kinetics data, the sericin-UM with 31% degree of modification was selected for subsequent cell culture experiments.

### 3.3. Microstructure of SerAte Hydrogels

**Figure 4:**
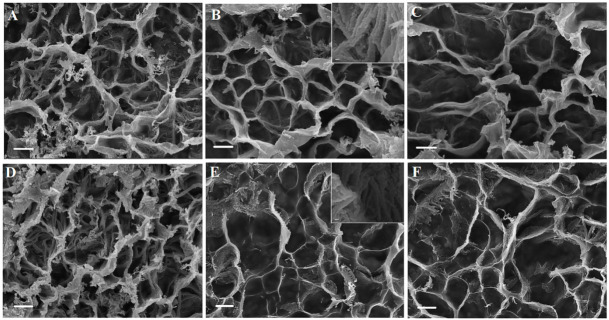
SEM images of lyophilized 15 wt% SerAte-UM hydrogels with degree of modification of 24% (**A**), 31% (**B**), and 40% (**C**) at 1100× magnification. SEM images of lyophilized 15 wt% SerAte-M hydrogels with degree of modification of 24% (**D**), 31% (**E**), and 40% (**F**) at 1100× magnification. The insets in (**B**–**E**) show higher magnification (3000×) SEM images of SerAte-UM and SerAte-M hydrogels, respectively, with 31% degree of modification. The scale bar in (**A**–**F**) is 10 µm and that in the insets of (**B**,**E**) is 0.5 µm.

The SEM images in Figure 4 show the microstructure of freeze-dried SerAte-M and SerAte-UM hydrogels at different degrees of modification at 1.1 K magnification. Overall, the SerAte hydrogels showed a stable, interconnected honeycomb microstructure with high porosity. The pore size of the hydrogels, averaged over at least 20 pores in the SEM images, was in the range of 39–54 μm with <5 µm honeycomb wall thickness. The average pore size of SerAte-M and SerAte-UM hydrogels increased with an increasing degree of modification, as shown in Table 1. For a given degree of modification, the SerAte hydrogels had slightly larger average pore sizes as compared to SerAte-M hydrogels (Table 1), which was attributed to the more hydrophilic ethyl urethane groups in SerAte-UM [42]. The inset images in Figure 4B,E show the fibrous nature of the assembled SerAte-M and SerAte-UM molecules in the pore wall of the hydrogels. High porosity and pore interconnectivity are crucial to homogenous cell seeding, balancing cell–cell versus cell–matrix interactions, nutrient and metabolite diffusion, and ECM production [43].

### 3.4. Compressive Modulus and Swelling of SerAte Hydrogels

**Figure 5:**
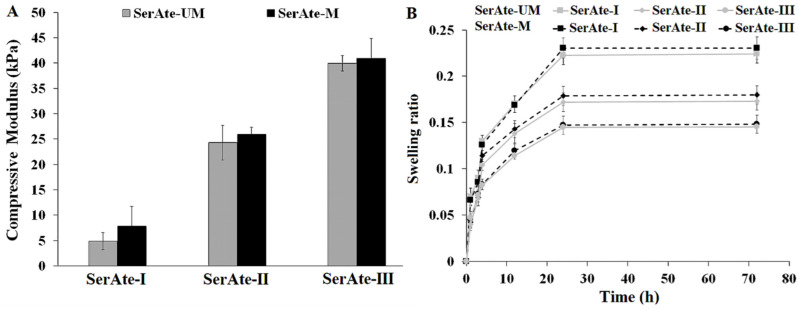
Effect of degree of modification on compressive modulus (**A**) and swelling ratio in PBS (**B**) of SerAte-UM and SerAte-M hydrogels (15 wt% SerAte) at ambient conditions. SerAte-I, SerAte-II, and SerAte-III correspond to SerAte hydrogels with 24%, 31%, and 40% degree of modification, respectively. The values are expressed as means ± SD for n = 3.

Hydrogel porosity and pore interconnectivity are directly related to water content and the extent of swelling, but inversely related to mechanical strength [43]. Figure 5A shows the effect of degree of modification on the compressive modulus of SerAte-M and SerAte-UM hydrogels. The compressive modulus of SerAte-M and SerAte-UM hydrogels increased with increasing degree of modification, which was attributed to the higher concentration of unsaturated methacrylate groups used in crosslinking. The compressive modulus of SerAte-M was slightly higher than that of SerAte-UM, which was attributed to the higher molecular weight of IEM compared to M (eight bonds in IEM versus three bonds in M after modification; see Figure 1A), but the difference was not statistically significant. For example, the compressive modulus of SerAte-M with 31% degree of modification was 23.7 ± 2.1 kPa whereas that of SerAte-UM was 20.4 ± 2.7 kPa. Figure 5B shows the effect of degree of modification on the swelling ratio of SerAte-M and SerAte-UM hydrogels as a function of incubation time. For a given incubation time, the swelling ratio of SerAte-M and SerAte-UM hydrogels decreased with increasing degree of modification. For a given degree of modification, the swelling ratio increased rapidly initially, followed by increasing at a slower rate to reach a plateau. For a given degree of modification and incubation time, the swelling ratio of SerAte-UM was slightly higher than that of SerAte-M, which was attributed to hydrogen bonding between water molecules and the urethane groups in SerAte-UM. The swelling ratio of SerAte hydrogels ranged from 1.5 to 2.25, which indicated that both hydrogels exhibited excellent hydrophilicity for cell encapsulation in tissue engineering [44].

### 3.5. Degradation and Permeability of SerAte Hydrogels

**Figure 6:**
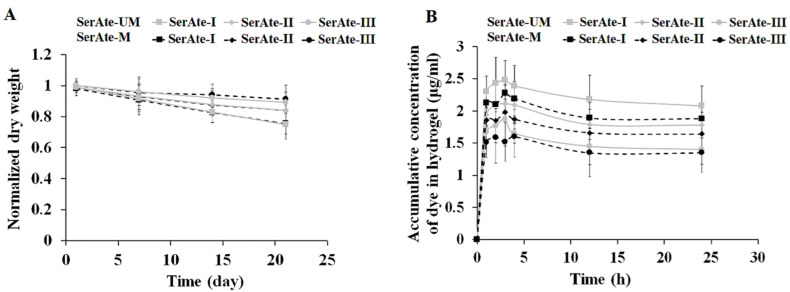
Effect of degree of modification on mass loss (**A**) and dye uptake (**B**) of SerAte-UM and SerAte-M hydrogels (15 wt% SerAte) in PBS at ambient conditions. The values are expressed as means ± SD for n = 3.

The effect of degree of modification on the fractional mass loss of SerAte-UM and SerAte-M hydrogels is shown in Figure 6A. For a given degree of modification and incubation time, the mass loss of SerAte-UM was slightly higher than that of SerAte-M, which was attributed to the slightly higher hydrophilicity of IEM compared to M, but the difference was not statistically significant. For a given time, the mass loss of SerAte-UM and SerAte-M hydrogels decreased with increasing degree of modification, which was attributed to the lower swelling ratio, higher crosslink density, or shorter chains between crosslinks at higher degrees of modification. The mass loss of SerAte-UM hydrogel was <25% after 21 days of incubation for all degrees of modification, indicating that these hydrogels possess sufficient stability for use as a cell carrier in tissue regeneration.

The permeability of SerAte hydrogels was measured by diffusion of fluoresceinamine isomer I dye from the medium and its accumulation in the hydrogel, and the results are shown as a function of incubation time in Figure 6B. An overshoot was observed in the dye uptake curves of SerAte hydrogels following the rapid initial increase, which was not observed in the curves for swelling ratio (Figure 5B). The overshoot was attributed to transient changes in the hydrogel microstructure due to physical crosslinking (via hydrogen bonding) coupled with the limited dye solubility in aqueous solution [45]. Dye uptake decreased with increasing degree of modification, which was attributed to the higher degree of crosslinking and the lower water uptake at higher degrees of modification. For a given degree of crosslinking and incubation time, the dye uptake of SerAte-UM hydrogel was significantly higher than that of SerAte-M, which was attributed to the differences in their swelling ratio (Figure 5B). The results of dye uptake studies demonstrate that SerAte-UM hydrogels are permeable to nutrients and growth factors in order to maintain the viability, function, and growth of encapsulated cells in hydrogel scaffolds.

### 3.6. Viability of MSCs Encapsulated in SerAte Hydrogels

**Figure 7:**
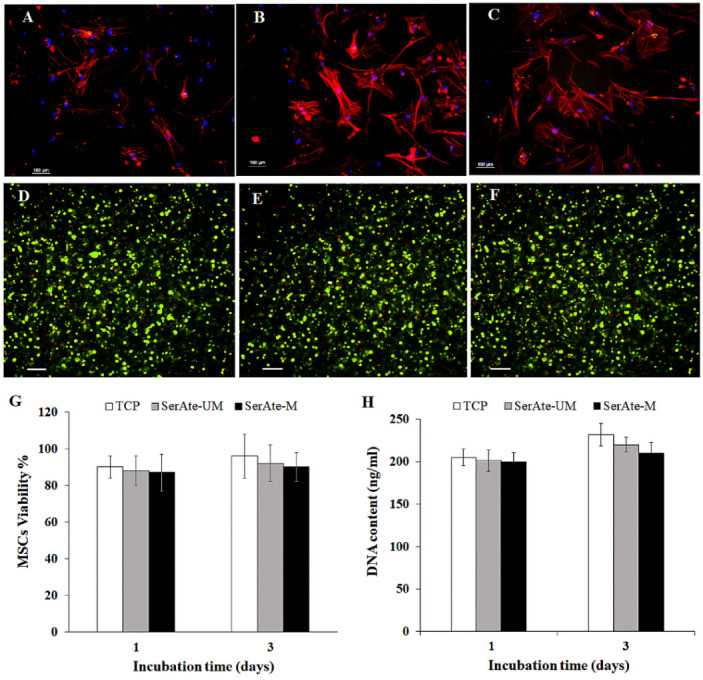
DAPI (blue) and phalloidin (red) stained images of hMSCs seeded on TCP (**A**), SerAte-UM (**B**), and SerAte-M (**C**) hydrogels after three days of incubation in basal medium. Live (green) and dead (red) stained images of hMSCs seeded on TCP (**D**) or encapsulated in 15% wt SerAte-UM (**E**) and SerAte-M (**F**) hydrogels with 31% degree of modification after three days of incubation. The scale bar in (**A**–**F**) is 100 μm. Percent viability (**G**) and dsDNA content (**H**) of hMSCs seeded on TCP and encapsulated in 15 wt% SerAte-UM and SerAte-M hydrogels with 31% degree of modification as a function of incubation time. Error bars correspond to mean ±SD for n = 3.

DAPI- and phalloidin-stained images of MSCs seeded on adherent polystyrene tissue culture plate (TCP) and SerAte-UM and SerAte-M hydrogels are shown in Figure 7A–C, respectively. Images B and C in Figure 7 show the elongated spindle-shape morphology of MSCs on SerAte-UM and SerAte-M hydrogels, consistent with previous reports on the morphology of MSCs on keratin-based hydrogels [9]. The SerAte hydrogels presumably facilitated cell attachment through serine-rich repetitive sequences [41]. The higher intensity of actin filament staining in Figure 7B indicated stronger interaction of surface receptors of MSCs with adhesion ligands on the surface of SerAte-UM hydrogel compared to TCP and SerAte-M hydrogel. Figure 7D–F show live (green) and dead (red) stained images of MSCs seeded on TCP, encapsulated in SerAte-UM and in SerAte-M hydrogels (15 wt% SerAte, 31% degree of modification), respectively, after three days of incubation in basal medium. The images show a high fraction of viable MSCs three days after encapsulation in SerAte hydrogels. It should be noted that TRITC-phalloidin, used in adhesion experiments (images A–C), stains the filamentous F-actin in the cell cytoplasm, thus imaging the spread morphology of the cells as delineated by actin filaments. By contrast, Calcein-AM, used in viability experiments (images D–F), permeates the cell membrane and is converted to fluorescent Calcein by reaction with intracellular esterase, thus imaging only cell viability by fluorescing green and not cell shape.

The fraction of viable encapsulated hMSCs, quantified by dividing the images into smaller squares and counting the number of live and dead cells, is shown in Figure 7G after one and three days of incubation. The fractions of viable cells encapsulated in SerAte-UM hydrogel were 88 ± 1% and 92 ± 2% after one and three days of incubation, respectively, and the fractions of viable cells encapsulated in SerAte-M hydrogel were 87 ± 3% and 89 ± 3%, respectively, consistent with the previously reported >92% viability of encapsulated fibroblasts in sericin hydrogels [31,46]. The number of cells encapsulated in the hydrogels with incubation time, quantified by measuring DNA content using PicoGreen assay, is shown in Figure 7H. The dsDNA content was normalized to the number of encapsulated MSCs at time zero. The number of viable cells in SerAte-UM and SerAte-M hydrogels increased slightly from day one to day three, indicating that these hydrogels support cell growth. Our results do not support the previous finding that sericin functionalization denatures or degrades sericin peptides to a lower viability of encapsulated cells [31].

## 4. Conclusions

Sericin extracted from silk cocoons was modified with 2-isocyanatoethyl methacrylate (IEM) to produce a photo-polymerizable sericin urethane methacryloyl (SerAte-UM) biopolymer and used as a hydrogel for encapsulation of MSCs. The secondary structure of the extracted sericin was not affected by functionalization with IEM. The SerAte-UM hydrogel was slightly more hydrophilic than the sericin methacryloyl (SerAte-M) hydrogel, which was attributed to the more hydrophilic nature of IEM compared to methacrylate in SerAte-M. The gelation time of SerAte-UM hydrogel decreased with increasing degree of modification. The photo-polymerized SerAte-UM hydrogel had a highly porous, fibrous, honeycomb microstructure with an average pore size in the 40–50 µm range. The compressive modulus, swelling ratio, and permeability of SerAte-UM hydrogel depended on the degree of modification of sericin, and the mass loss in aqueous solution was <25% after 21 days of incubation. The experimental results demonstrate that the SerAte-UM hydrogel is promising as a matrix for the encapsulation and delivery of stem cells for reconstruction of injured tissues.

## Figures and Tables

**Table 1:** The degree of methacrylation of sericin as a function of molar ratio of IEM to serine or MA to serine (IEM/MA:serine).

Group	SerAte-I	SerAte-II	SerAte-III
IEM/MA:sericin molar ratio	7:1	12:1	20:1
Modification %	24 ± 2	31 ± 5	40 ± 3
Pore size (µm^2^)	41.1 ± 16.6	49.6 ± 25.7	53.5 ± 17.9
	39.9 ± 15.8	46.7 ± 23.5	52.4 ± 15.8

## Data Availability

The data presented in this study are available on request from the corresponding author. The data are not publicly available due to protection of intellectual property.
